# The El Paso Meeting

**Published:** 1901-11

**Authors:** 


### TEXAS MEDICAL JOURNAL.

AUSTIN, TEXAS.

A MONTHLY JOURNAL OF MEDICINE AND SURGERY.

EDITED AND PUBLISHED BY
F. E. DANIEL, M. D.

ASSOCIATE EDITOR:
WITTEN BOOTH RUSS, M. D.,
San Antonio, Texas.

Published Monthly at Austin, Texas. Subscription price $1.00 a year in advance.

Eastern Representative: John Guy Monihan, St. Paul Building, 220 Broadway, New York City.

Official organ of the State Association of Health Officers, the West Texas Medical Association, the Houston District Medical Association, the Austin District Medical Society, the Brazos Valley Medical Association, the Galveston County Medical Society, and several others.

### THE EL PASO MEETING.

As is known to all readers of the JOURNAL, the Texas State Medical Association elected to meet next April at El Paso. After the meeting at Galveston (April last) the Red-Back expressed itself quite freely to the effect that the selection was an unfortunate one, in fact, a great mistake. Look at the map. El Paso is at the extreme western border of the State, and is 650 miles west of Fort Worth, and about the same distance, or further, from San Antonio. To reach El Paso via Fort Worth, a desert of dust and sand has to be crossed. In other words, it is a very disagreeable trip. The distance, the time required to make the trip to and fro, the four days of the meeting, the cost of the trip—all combine to keep members at home,—and the meeting, I much fear, will be a failure in point of numbers. Take the Palestine section,—in which there is a large and flourishing district medical society, most of the members being old and zealous members of the State Association. It is about 275 miles from Palestine to Fort Worth by the nearest route, making a total distance of nearly 1000 miles. It is simply prohibition to the doctors of East Texas.

There is talk of a ten dollar rate. Granted it is obtained,—and I don't believe it will be,—figure on the cost of the trip in time and money, outside of the railroad fare, and see if it is reasonable to expect a doctor from the eastern part of the State to attend the meeting?

I know, from conversation and correspondence with many members, that there is great dissatisfaction because of these things, and I have been informed that an organized movement will be made to get the place of meeting changed. It is contemplated to get a meeting of the officers at some central point soon to take action on the matter. I suggest that at the fall meeting of the several district medical societies a memorial from each be sent up to the President asking that he take steps to change the place of meeting to some point accessible to the rank and file of the Association. The change should be made before the gentlemen of the Arrangement Committee at El Paso go to any expense on account of the expected meeting.

It is of the greatest importance that the April meeting shall be full and representative. The proposed changes in the constitution are to be acted on, and every member should have a voice in the matter.

It is contended that El Paso is the only city that extended an *invitation* to the Association. The Association duly appreciates the courtesy, but the Association should not stand back and wait for an invitation. It should meet where it is most convenient to the majority of its members to meet; for the best interest of the Association. An invitation carries with it an obligation to entertain the members. The Association should not expect it. Political conventions are not invited nor entertained; why should medical conventions expect it or wait for it, or accept it? Let us meet at Dallas, or Fort Worth, or Austin, and let every man pay his own expenses and the treasury the hall rent and other expenses. I hope the medical societies this month will take the matter up.

